# Pandemic *Vibrio parahaemolyticus* O3:K6 on the American continent

**DOI:** 10.3389/fcimb.2013.00110

**Published:** 2014-01-02

**Authors:** Jorge Velazquez-Roman, Nidia León-Sicairos, Lucio de Jesus Hernández-Díaz, Adrian Canizalez-Roman

**Affiliations:** ^1^School of Medicine, Autonomous University of SinaloaCuliacan, Mexico; ^2^Pediatric Hospital of SinaloaCuliacan, Mexico; ^3^Programa Regional Para el Doctorado en Biotecnología, FCQB-UASCuliacan, México; ^4^The Sinaloa State Public Health Laboratory, Secretariat of HealthCuliacan, Mexico

**Keywords:** American continent, pandemic clone, serotypes, *Vibrio parahaemolyticus*, biosurveillance, public health

## Abstract

*Vibrio parahaemolyticus* is one of the most important seafood-borne bacterial in recent years and is the leading causal agent of human acute gastroenteritis, primarily following the consumption of raw, undercooked or mishandled marine products. Until 1996, infections caused by *V. parahaemolyticus* were generally associated with diverse serovars. However, in February 1996, a unique serovar (O3:K6) of *V. parahaemolyticus* with specific genetic markers (*tdh*, *toxRS/New* and/or *orf8*) appeared abruptly in Kolkata, India. In subsequent years, O3:K6 isolates similar to those isolated in Kolkata have been reported from food borne outbreaks in Southeast Asia, as well as in the Atlantic and Gulf coasts of the United States (U.S). More recently, there have been reports in Europe, Africa and Central and South America. Specifically, in the American continent, some countries have reported cases of gastroenteritis due to the pandemic O3:K6 strain and its serovariants; the pandemic strain was first detected in Peru (1996, >100 cases), subsequently spreading to Chile in 1998 (>16,804 human cases), to the U.S. in 1998 (>700 cases), to Brazil in 2001 (>18 cases) and to Mexico in 2004 (>1200 cases). The arrival of the pandemic clone on the American continent may have resulted in a significant shift on the epidemic dynamics of *V. parahaemolyticus*. However, although O3:K6 is the predominant serovar of the recognized clinical strains in some countries in the Americas, a decrease in clinical cases caused by O3:K6 and an increase in cases associated with a new serotype (O3:K59, Chile) have been recently reported. The emergence and worldwide dissemination of O3:K6 and other pandemic strains since 1996 have come to represent a threat to public health and should concern health authorities. This review focuses on the presence, distribution and virulence factors of the *V. parahaemolyticus* O3:K6 pandemic clone and its serovariants in clinical and environmental strains on the American continent.

## Introduction

*Vibrio parahaemolyticus* is a Gram-negative halophilic, mesophilic, non-spore forming, curved rod-shaped bacterium that naturally inhabits marine and estuarine environments worldwide (Baumann et al., 1984). It is commonly found free swimming, attached to underwater surfaces, and commensally associated with various species of shellfish (McCarter, 1999). *V. parahaemolyticus* has recently been recognized as one of the most important foodborne pathogens and as the leading causal agent of human acute gastroenteritis, primarily following the consumption of raw, undercooked or mishandled seafood and marine products (Okuda et al., 1997; Bag et al., 1999; Depaola et al., 2000; Wong et al., 2000; Ansaruzzaman et al., 2005; Martinez-Urtaza et al., 2005; Cabanillas-Beltran et al., 2006; Su and Liu, 2007; Pal and Das, 2010; Velazquez-Roman et al., 2012). The halophilic bacteria was first identified as a cause of food-borne illness during the fall of 1950 within the southern suburbs of Osaka, Japan, where an outbreak of acute gastroenteritis following the consumption of semidried juvenile sardines sickened 272 and killed 20 individuals (Fujino et al., 1953). *V. parahaemolyticus* causes two additional major clinical pathologies wound infections and septicemia. The most common syndrome is gastroenteritis, the symptoms of which include diarrhea with abdominal cramps, nausea, vomiting, headache, chills and low-grade fever (Honda and Iida, 1993). The associated diarrhea is occasionally bloody, characterized by reddish watery stools (Qadri et al., 2003) that are unlike those observed in cases of dysentery caused by *Shigella* species or in cases of amoebiasis. The mean incubation period for *V. parahaemolyticus* infection is 15 h (range: 4–96 h) (Joseph et al., 1982). The illness is self-limiting and of moderate severity, lasting an average of 3 days in immunocompetent patients (Yeung and Boor, 2004). Because of its self-limiting nature, most cases of infection by *V. parahaemolyticus* can be can be treated by oral rehydration, alone. Occasionally, treatment with antibiotics such as doxycycline, ciprofloxacin, or erythromycin is necessary (Qadri et al., 2003). The infection can be fatal for immunocompromised patients or for those with a preexisting medical condition, such as liver disease or diabetes. Although the overall pathogenetic mechanism of marine *Vibrio* species such as *V. parahaemolyticus* is not completely understood, these pathogens are known to produce various extracellular products, some of which are known pathogenicity factors (Hasegawa and Hase, 2009). Potentially virulent strains are commonly distinguished from virulent strains by the presence of two hemolysins, namely the thermostable direct hemolysin (*tdh*) and the Tdh-related hemolysin (*trh*), which exhibits 68% homology with the *tdh* gene (Zhang and Austin, 2005), and plays a significant role in precipitating the disease (Takeda, 1983; Shirai et al., 1990; Bej et al., 1999). Pathogenic *V. parahaemolyticus* can produce either TDH, TRH, or both (Honda and Iida, 1993; Nishibuchi and Kaper, 1995). An isolate that produces TDH is referred to as Kanagawa phenomenon (KP)-positive and can be identified by β hemolysis on a special agar known as Wagatsuma blood agar (Pal and Das, 2010; Canizalez-Roman et al., 2011; Velazquez-Roman et al., 2012). PCR analysis targeting the *tdh* gene encoding TDH hemolysin and/or the trh gene encoding TRH hemolysin have been widely used for the identification of pathogenic isolates of *V. parahaemolyticus* (Shirai et al., 1990; Yoh et al., 1995).

## Pathogenic *V. parahaemolyticus*

Although the presence of *V. parahaemolyticus* is extensive in marine, estuarine and riverine environments, not all strains of *V. parahaemolyticus* are considered pathogenic. *Vibrios* cultured from environmental samples commonly lack the genes coding for proteins with associated pathogenic functions in humans and marine animals (e.g., *tdh* in *V. parahaemolyticus*) (Deepanjali et al., 2005; Canizalez-Roman et al., 2011; Gutierrez West et al., 2013). However, few studies have reported the presence of the *tdh* and *trh* genes in *V. parahaemolyticus* strains of environmental origin; only 0–6% of the samples analyzed from the coasts of the U. S. (Kaysner et al., 1990; Depaola et al., 2000), Europe (Hervio-Heath et al., 2002), and Asia contained the *tdh* and/or *trh* genes (Vuddhakul et al., 2000; Wong et al., 2000; Alam et al., 2002). Most of the environmental strains are known to be KP-negative, and only 1–2% of the samples are KP-positive (Joseph et al., 1982; Nishibuchi and Kaper, 1995). In molecular epidemiological studies, up to 90% of the isolated clinical strains have been found to possess the *tdh* and/or *trh* gene (the serotype O3:K6 strain has increased in prominence) (Okuda et al., 1997; Garcia et al., 2009; Chao et al., 2010; Velazquez-Roman et al., 2012), whereas their presence in environmental isolates is rare (Shirai et al., 1990; Depaola et al., 1992; Nishibuchi and Kaper, 1995; Islam et al., 2004; Yeung and Boor, 2004; Nair et al., 2007; Chao et al., 2010). However, in limited areas of Mexico and the U.S., increased proportions (48–52%) of strains with pathogenic markers (*tdh* and/or *trh*) have been detected in environmental isolates (Velazquez-Roman et al., 2012; Gutierrez West et al., 2013). The increased rate of detection of pathogenic *V. parahaemolyticus* within the marine environment might be attributed to a decreased prevalence of total *V. parahaemolyticus* rather than to an increased abundance of pathogenic strains (Velazquez-Roman et al., 2012).

Currently, a comprehensive understanding of the ability of this organism to cause disease remains incomplete. Although the isolates that do not contain *tdh* or those that exhibit a deletion in *tdh* are still cytotoxic to host cells, the overall mechanism underlying the pathogenesis of these *tdh*-negative strains remains unclear (Boyd et al., 2008). Isolates TDH positive has been shown to have hemolytic, enterotoxic, cardiotoxic, and cytotoxic activities (Nishibuchi et al., 1992; Raimondi et al., 2000). These cellular events are caused by bacterial effector proteins, include cytolysins, proteases, lipases, siderophores, exopolysaccharides, and effectors which are delivered into intestinal cells (enterotoxic) that directly modulate the activities of host cell proteins and are secreted and translocated into host cells via type III secretion systems (T3SS) (Hiyoshi et al., 2010, 2011; Matsuda et al., 2012) and recently type VI secretion systems (T6SS) (Yu et al., 2012; Salomon et al., 2013).

The complex interactions between pathogens and the hosts that they infect have long been believed to represent the primary driving forces that determine the strategies used by bacteria to counter host defenses. However, new evidence suggests that the external environment, including other hosts, might play a greater role in the evolution of certain pathogens than previously expected (Wilson and Salyers, 2003). Thus, the pandemic strains that exhibit certain biological characteristics, such as elevated toxin production or the ability to survive within the natural environment, might provide further insights into the mechanisms underlying the emergence and spread of these strains (Wong et al., 2000). Factors that affect the incidence and distribution of *V. parahaemolyticus* in the environment are known to include the water temperature, salt and oxygen concentrations, interaction with plankton, presence of sediment, organic matter in suspension, fish, and seafood, as well as the incorporation and tidal action of estuarine waters (Cabrera-Garcia et al., 2004). However, how these strains disseminate worldwide remains unknown.

## *vibrio parahaemolyticus* O3:K6 in the world

Until 1996, infections caused by *V. parahaemolyticus* were usually associated with diverse serovars (for example, O1:K38, O3:K29, O4:K8, O3:K6, O2:K3, O4:K8, and other serotypes) (Okuda et al., 1997; Wong et al., 2000) and exhibited a localized distribution, emerging in different areas of the world only during the warmer months of the year. However, the epidemiology of *V. parahaemolyticus* changed drastically in February 1996, when an atypical increase in *V. parahaemolyticus* infections occurred in Kolkata, a city in the northeastern part of India, resulting in hospitalized patients with diarrheal infections. These infections were linked to strains that belonged to a clonal group of the O3:K6 serotype, which exhibited the specific genetic markers *tdh*, *toxRS/New* and *orf8*. These strains had not been previously isolated, but subsequent surveillance in Kolkata identified this serotype as responsible for 50–80% of the infections during the following months. This clone rapidly spread throughout the majority of Southeast Asian countries within a single year (Okuda et al., 1997; Chowdhury et al., 2000). During subsequent years, O3:K6 isolates similar to those isolated in Kolkata were reported from foodborne outbreaks and from sporadic cases in the Atlantic and Gulf coasts of the U.S. (Okuda et al., 1997; Chowdhury et al., 2000; Matsumoto et al., 2000). Recently, there have been similar reports in Europe (Martinez-Urtaza et al., 2005), Africa (Ansaruzzaman et al., 2005), North, Central and South America (Daniels et al., 2000a; Gonzalez-Escalona et al., 2005; Velazquez-Roman et al., 2012). The widespread occurrence of this single *V. parahaemolyticus* serotype had not previously been reported; thus, it became evident that a pandemic strain had emerged.

Currently several serovariants of the pandemic strains of O3:K6, included 14 different serotypes (O1:K25, O1:K41, O1:K56, O1:KUT, O3:K6, O3:K58, O3:K68, O3:K75, O4:K8, O4:K12, O4:K68, O4:KUT, O5:KUT, and OUT:K6), although only O3:K6, O4:K68, O1:K25, O1:26, and O1:KUT have been recognized as the predominant group responsible for most outbreaks since 1996. These serotypes have been identified as pandemic clones (Okuda et al., 1997; Matsumoto et al., 2000; Okura et al., 2003; Chowdhury et al., 2004a; Ansaruzzaman et al., 2005; Mahmud et al., 2007; Nair et al., 2007; Velazquez-Roman et al., 2012). The acquisition of additional serotypes of the pandemic strain may be a selective response to host immunological pressure (Chowdhury et al., 2004b). Molecular studies based on pulsed-field gel electrophoresis and arbitrarily primed PCR (APPCR) have indicated that the pandemic strains exhibit almost identical fragment patterns (Depaola et al., 2000; Parvathi et al., 2006). Notably, since 1996, a filamentous phage (*f*237) (Nasu et al., 2000; Iida et al., 2001) has been reported to be associated with the pandemic isolates of *V. parahaemolyticus* (Nasu et al., 2000; Iida et al., 2001). Therefore, from a single O3:K6 serotype, other serotypes that exhibit identical genotypes and molecular profiles to the O3:K6 isolates have emerged. These new serotypes have been collectively called the “serovariants” of O3:K6 isolates (Matsumoto et al., 2000). Generally, pandemic strains belong to the O3:K6 serotype and contain the *orf8* gene. The *orf8* gene is believed to encode an adherence protein that increases the ability of *V. parahaemolyticus* to adhere to host intestinal cells or to the surfaces of marine plankton (Nasu et al., 2000). Furthermore, several studies have reported that the *toxRS* operon of the pandemic strains contains a unique sequence (*toxRS/new*) that encodes for transmembrane proteins involved in the regulation of virulence-associated genes (Chowdhury et al., 2000; Matsumoto et al., 2000; Okura et al., 2003). Characteristics of the O3:K6 pandemic clone isolates include the O3:K6 antigens, a distinctive *toxRS* sequence (*toxRS/new*) (Matsumoto et al., 2000), *orf8* (Nasu et al., 2000) and t*dh* genes, and the absence of the *trh* gene that is found in some pathogenic strains. Although several polymerase chain reaction (PCR)-based methods targeting *toxRS/new*, group-specific PCR (GS-PCR) or *orf8*—have been developed, the presence of the *toxRS/new* gene is necessary but not always sufficient, whereas the *orf8* gene is sufficient but not always necessary for the detection of pandemic strains (Chowdhury et al., 2000; Okura et al., 2003). In general, however, based on the presence or absence of virulence genes, an isolate possessing both *tdh* and *toxRS/new* genes can be considered a pandemic strain (Okura et al., 2003). One of the other virulence genes, *trh*, is not specific to pandemic strains and is rarely present in environmental strains compared to clinical strains (Depaola et al., 2000; Parvathi et al., 2006). Pathogenic strains feature *tdh*- and/or *trh*-positivity, whereas non-pathogenic strains are characteristically *tdh*- and *trh*-negative. Some clinical isolates have been found to contain both *tdh* and *trh* genes, whereas most environmental isolates possess neither (Shirai et al., 1990; Baba et al., 1991; Kishishita et al., 1992; Xu et al., 1994).

## Pandemic O3:K6 clone on the american continent

**Figure 1:**
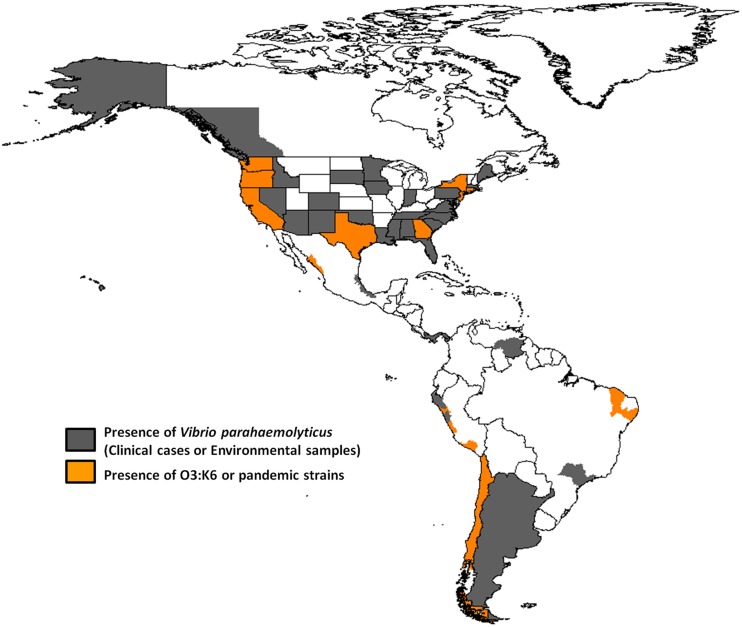
**Map showing the presence of *Vibrio parahaemolyticus* and the dissemination of pandemic O3:K6 clone throughout the American continent**.

The routes and mechanisms resulting in the dissemination of the pandemic strain are still unclear. Moreover, the epidemiology of *V. parahaemolyticus* infections remains poorly understood. Recently, there have been occurrences of other extraordinary events regarding pathogenic *Vibrio* species. The transmission and epidemiology of *V. parahaemolyticus* infections in places such as Kolkata, India, and Bangladesh are entirely distinct from the rest of the world because seafood is never eaten raw, and freshwater fish are preferred over seawater fish by the local population (Sarkar et al., 1985). However, in February 1996, the O3:K6 serotype emerged in Kolkata with identical genotypes (*tdh* positive and *trh* negative) and profiles that were indistinguishable by molecular subtyping techniques (Okuda et al., 1997). In subsequent years, isolates similar to those from Kolkata were reported from foodborne outbreaks and from sporadic cases in several sites worldwide (Daniels et al., 2000a; Matsumoto et al., 2000; Ansaruzzaman et al., 2005; Gonzalez-Escalona et al., 2005; Martinez-Urtaza et al., 2005; Leal et al., 2008; Velazquez-Roman et al., 2012), particularly during the warmer months of the year. Subsequently, the pandemic clone began global dissemination, with many outbreaks. In particular, some countries of the American continent have reported cases of gastroenteritis due to the pandemic O3:K6 strain and its serovariants, where the pandemic strain was first detected in Peru (>100 cases at approximately the same time that it caused an outbreak in Calcutta in February 1996) (Gil et al., 2007). The O3:K6 strain subsequently spread to Chile in 1998 (>16,804 human cases), to the U.S. in 1998 (>700 cases), to Brazil in 2001 (>18 cases) and to Mexico in 2004 (>1200 cases) (Figure 1 and Table 1).

**Table 1:** **Presence of *Vibrio parahaemolyticus* and the pandemic O3:K6 clone in clinical cases and the environment on the American continent**.

**Country (year of isolation)**	**Source of isolates and serovars collected (origin type)**						**Reference**
	**(No. Cases) Clinical**	Pandemic ()^*^	**Other serotypes**	**(No. Samples) Environmental**	Pandemic ()^*^	**Other serotypes**	
Peru (1993–2007)	(424) Gastroenteritis	O3:K6 (1996)	O1:K33, O2:K3, O3:K30, O3:K58	Fish	O3:K6 (1996)	No data available	Gil et al., 2007
		O3:K68	O3:K68, O4:K8, O4:K12, O4:K55				
		O3:K58	O6:K18, O6:K46, O11:K15, O1:KUT, O2:KUT	Shrimp			Gavilan et al., 2013
		OUT:K6	O5:KUT, O6:KUT, O11:KUT, OUT:K3				
			OUT:K6, OUT:K8, OUT:K46, OUT:KUT				
Chile (1998–2013)	(>16000) Gastroenteritis	O3:K6 (1998)	O1:K56, O3:K6, O3:K59, O4:K12	Shellfish	O3:K6 (1998)	O3:KUT	Gonzalez-Escalona et al., 2005
			O10:K20, O1:KUT, O3:KUT, OUT:KUT				Garcia et al., 2009
							Harth et al., 2009
United States of America (1973–2012)	6159	O3:K6 (1998)	O1:K20, O1:K25, O1:K33, O1:K43	Shellfish		No data available	Daniels et al., 2000a,b
	Gastroenteritis		O1:K56, O3:K39, O3:K5, O3:K56				Depaola et al., 2000
	Wound infections Septicemia		O4:K4, O4:K8, O4:K9, O4:K12				McLaughlin et al., 2005
			O4:K13, O4:K34, O4:K37, O4:K42				Newton et al., 2012
			O4:K53, O4:K63, O5:K17, O5:K30	Fish			
			O5:K47,O6:K18, O8:K41, O8:K70				
			O1:KUT, O3:KUT, O4:KUT, O5:KUT				
			O6:KUT, O8:KUT, O10:KUT, O11:KUT				
Brazil (1989–2002)	(39) Gastroenteritis	O3:K6 (2002)	O1:K56, O3:K5 O3:K6, O3:K58	(23) Shellfish		No data available	Hofer, 1983
		O3:KUT	O3:KUT, O4:K4, O4:K10, O4:K12 O4:K53, O4:KUT, O5:KUT, O10:KUT				Magalhaes et al., 1991a,b, 1992
							Leal et al., 2008
							Sobrinho Pde et al., 2010
Mexico (2001–2010)	(>1379) Gastroenteritis	O3:K6 (2004)	O1:KUT, O1:K9, O1:K33, O3:K58	(190) Shellfish	O3:K6 (2004)	O1:K25, O1:K26, O1:K32, O1:K33	Cabrera-Garcia et al., 2004
		O3:KUT	O3:K68, O4:KUT, O4:K8, O4:K10	Fish	O3:KUT	O1:K41, O1:K56, O2:K28, O3:k4	Cabanillas-Beltran et al., 2006
			O4:K12, O4:K63, O5:K15, O5:K17	Seawater	O10:KUT	O3:K6, O3:K29, O3:K32, O3:K33	
			O6:K46, O8:K20, O10:K52, O1:KUT			O3:K58, O3:K59, O3:K68, O4:K10	Velazquez-Roman et al., 2012
						O4:K12, O4:K53, O4:K68, O5:K15,	
						O5:K17, O5:K68, O6:K6, O6:K46	
			O4:KUT, O5:KUT, O8:KUT, O11:KUT	Sediment		O8:K8, O8:K20, O8:K21, O10:K52,	
			OUT:KUT			O1:KUT, O2:KUT, O3:KUT, O4:KUT	
						O5:KUT, O6:KUT, O7:KUT, O10:KUT	
						O11:KUT, OUT:K8, OUT:KUT	
Canada (1997–2006)	(>200) Gastroenteritis	No data available		Shellfish		No data available	FAO/WHO, 2011
							Khaira and Galanis, 2007
Costa Rica (1985–2002)				(77) Shellfish		No data available	Garcia Cortes and Antillon, 1990
				Sediment			Díaz, 2002
Puerto Rico (1986–1988)				Shellfish		No data available	Rivera Susan et al., 1989
				
Panama (1974–1975)	(>1) Gastroenteritis	No data available		Shrimp		No data available	Kourany et al., 1974; Kourany and Vasquez, 1975
Venezuela (2011)				Shellfish		No data available	Grau et al., 2004
				Prawn			Muñoz et al., 2008
				Coral			Garcia-Amado et al., 2011
Argentina (1977)				Shellfish		No data available	Casellas et al., 1977

* Year of isolation of O3:K6 pandemic clone.

### Peru

Peru was the first country where the *V. parahaemolyticus* O3:K6 clone was found in marine food products (raw fish or seafood marinated with lemon juice, popularly called “cebiche”) (Guevara Duncan and Del Carpio, 1989) and is where the strain was first associated with gastroenteritis on the American continent (Ibarra and Alvarado, 1999; Gil et al., 2007). Case records from 1994–1996 reflect occasional scattered infections restricted to the warmest months of the year (Martinez-Urtaza et al., 2008). Gil et al. found that the first O3:K6 strains were isolated in February 1996 in Trujillo city, indicating that the pandemic strain was present in Peru at approximately the same time that it caused the outbreak in Calcutta in February 1996 (Gil et al., 2007). In 1997, a sudden increase in the number of O3:K6 *V. parahaemolyticus* cases was observed in Chiclayo, Department of Lambayeque (in northern Peru) in July, which lasted for 10 months with 2 peaks. Infections were observed along the entire coastline of Peru and spread more than 1500 km within a 4-month period (Martinez-Urtaza et al., 2008). The infections spread in a constant southward direction, affecting the Department of Cajamarca in August, La Libertad in September, and Huaraz in October, finally reaching the southern Peruvian border in November—at the same time as the emergence of *V. parahaemolyticus* illnesses within the northern Chilean city of Antofagasta (Gonzalez-Escalona et al., 2005; Martinez-Urtaza et al., 2008). In a retrospective analysis of data on *V. parahaemolyticus* in Peru, Gil et al., indicated that although the first O3:K6 strain was isolated in February 1996 in Trujillo city, the first cluster of cases caused by the pandemic serotype occurred in Lima in 1998, when 27 strains were isolated from hospitalized cases in a short span of time in one hospital (Gil et al., 2007).

Interestingly, the non-pandemic O4:K8 and O4:K55, and O5:KUT serotypes predominated in 1994 and 1996 in Trujillo, and the infections were characteristically related to self-limiting outbreaks that were detected along the coastal regions during the summer months (Martinez-Urtaza et al., 2008). The serovar dominance, particularly that of O4:K8, abruptly changed during the winter of 1997, with the emergence of infections caused by strains belonging to the pandemic clone (Gavilan et al., 2013). Additional strains belonging to the O3:K68, O3:K58, and OUT:K6 serotypes were isolated in Lima and Arequipa in 1997 and found to be positive for *Orf8* and *GS-PCR*, indicating that they were also pandemic strains (Gil et al., 2007). From 1998 to 2002, pandemic strains (O3:K6 and others) were detected in Lima and Trujillo (Gil et al., 2007). Due to the environmental nature of *V. parahaemolyticus*, this type of change in population dominance and the subsequent population admixture might be expected to lead to an unpredictable impact on the populations of pathogenic *V. parahaemolyticus* in Peru (Gavilan et al., 2013). Finally, an undefined pattern of serovar dominance was detected after the emergence of the pandemic clone in 1997–1998. In this post-pandemic period, pandemic strains were identified together with O4:K8 strains, as well as multiple serovars that had not been previously detected (O1:K33, O1:KUT, O3:K30, O3:K58, O3:KUT, O5:KUT, O6:KUT, and OUT:KUT) (Gavilan et al., 2013). One particular feature of the epidemiology of *V. parahaemolyticus* in Peru after the emergence of the pandemic clone was the sudden appearance of diverse serovars that were not previously detected (Gavilan et al., 2013). Recently, in 2009, an outbreak of diarrhea attributed to infection by *V. parahaemolyticus* of the O3:K59 serotype with pandemic traits was reported in Peru in the Lambayeque, Piura and Lima regions (Zamudio et al., 2011) (Figure 1 and Table 1).

The epidemic dissemination of the O3:K6 pandemic clone along the coast of Peru appears to correspond to the expansion and dynamics of the poleward propagation and receding of tropical waters associated with the 1997 El Niño event (Martinez-Urtaza et al., 2008; Ansede-Bermejo et al., 2010). The arrival of the pandemic clone to Peru in 1997 resulted in a significant shift in the epidemic dynamics of *V. parahaemolyticus* within the region, replacing the seasonal and local self-limited infections attributed to native genetic groups with the generalization of infections exclusively caused by the pandemic strains across the country and possibly on the American continent (Gil et al., 2007; Martinez-Urtaza et al., 2008; Ansede-Bermejo et al., 2010; Velazquez-Roman et al., 2012). Recently, the Instituto Nacional de Salud del Perú (INS) became a member of the WHO Global Foodborne Infections Network (WHO-GFN) and of the PulseNet Latin American and Caribbean Net (PN-AL & C), with whom it shares the genetic profiles of the isolated pathogenic strains, making it possible to compare the genotypes of similar strains found in different countries and to identify the occurrence of epidemic outbreaks within the region (Zamudio et al., 2011).

### Chile

Over the past decade, trends in *V. parahaemolyticus* illnesses associated with molluscan shellfish have taken unexpected and unprecedented turns. Pandemic *V. parahaemolyticus* has now spread to at least 5 continents and has caused repeated annual outbreaks in Chile, where *V. parahaemolyticus* was not even considered a problem when this risk assessment began (Gonzalez-Escalona et al., 2005). The spread of the pandemic clonal O3:K6 complex reached the Southern Hemisphere as early as 1998, two years after the strain abruptly appeared in Calcutta, India, in 1996 (Gonzalez-Escalona et al., 2005). An outbreak that occurred primarily within the northern city of Antofagasta in Chile from November 1997 to March 1998 caused >300 clinical cases (Cordova et al., 2002). This city is near the border with Peru. A second outbreak affecting approximately 1500 individuals occurred from January to March 2004, predominantly in Puerto Montt, a region that is known for cold coastal waters. The outbreak in Puerto Montt was likely triggered by higher-than-normal temperatures during the summer months within the region, which is generally cool during all seasons (Gonzalez-Escalona et al., 2005). This last outbreak had important economic and social repercussions because this region is one of the main shellfish-producing areas in Chile (Gonzalez-Escalona et al., 2005). The specific infection dynamics found in Chile, where infections associated with the pandemic clone started in 1997–1998 and subsequently in 2004 and where the pandemic clone has been predominant among clinical isolates, clearly contrasts with the epidemiological trend of arrival and the rapid decline of pandemic *V. parahaemolyticus* infections in Peru.

During the summer of 2005, one of the outbreaks in Chile was high-magnitude, causing >11000 cases. This is the largest outbreak documented for this pathogen, most of which have been associated with consumption of clams and mussels but not oysters, which reflects the pattern of shellfish consumption in Chile (Gonzalez-Escalona et al., 2005; Fuenzalida et al., 2006; Cabello et al., 2007). The large epidemics of diarrhea observed during the last two austral summers in Puerto Montt, Chile, appears to be directly related to the introduction of the O3:K6 serovar pandemic strain to this region (Gonzalez-Escalona et al., 2005; Fuenzalida et al., 2006). Until 2006, every analyzed case in Chile was caused by the serovar O3:K6 pandemic strain (Olea et al., 2005; MINSAL, 2013). In the summer of 2007, among the 477 cases reported, 73% corresponded to the pandemic complex (Olea et al., 2005; MINSAL, 2013), among which 40% of the clinical cases were associated with the O3:K59 serotype and 27% were unrelated to the pandemic strain (Garcia et al., 2009; Harth et al., 2009). In the summer of 2008, there was an unexpected increase from 477 to 1143 clinical cases that were associated with the pandemic strain serotype O3:K6 (98%) (Garcia et al., 2009; Harth et al., 2009). According to data from the Ministery of Health of Chile, more than 3640 cases of gastroenteritis were reported in 2008, although the reports do not specify the serotype of the *V. parahaemolyticus* isolates (MINSAL, 2013). In 2009, clinical cases decreased to 441, and only 64% were related to the pandemic strain; the remaining cases were related to non-pandemic *tdh*- and *trh*-negative strains (Garcia et al., 2009). Gradually, outbreaks have decreased to fewer than 10 cases in 2010 and 2011 (Garcia et al., 2013). In Chile, the pandemic strain had become a relatively stable bacterial subpopulation of the diverse *V. parahaemolyticus* population that is present in shellfish (Garcia et al., 2009).

This cycle of proliferation and the disappearance of pathogenic strains might be the result of a combination of biological and physical environmental factors, including temperature, bacterial solar exposure and interaction with bacteriophages, which has been demonstrated in other regions of the world (Kaneko and Colwell, 1973; Kaneko, 1977; Kaysner et al., 1990; Garcia et al., 2013). However, the significant mechanisms of transmission remain poorly defined.

### United states of america

In the U.S., *Vibrio* infections result in an estimated 80000 illnesses, 500 hospitalizations, and 100 deaths every year (Baba et al., 1991). The United States has been prone to *V. parahaemolyticus* infections since the identification of this pathogen in 1971 in Maryland, where steamed crabs and crabmeat were implicated in the outbreaks (Dadisman et al., 1972). A total of forty *V. parahaemolyticus* outbreaks associated with consumption of raw or cooked seafood cross-contamination occurred in the U.S. between 1973 and 1998 (Daniels et al., 2000a). One such report was of sporadic *Vibrio* infections in two coastal areas of Louisiana and Texas in the U.S. between 1992–1993, when crayfish consumption was reported by 50% of the individuals infected by *V. parahaemolyticus* (Bean et al., 1998). Furthermore, three large multistate outbreaks occurred in 1997 and 1998. An outbreak linked to the consumption of raw or undercooked shellfish harvested from the waters off the coasts of California, Oregon, Washington, and British Columbia resulted in 1200 illnesses in 7 states and Canada (CDC, 1998), between July to August 1997. The most common *V. parahaemolyticus* serotypes isolated from patients involved in this outbreak were O4:K12 and O1:K56. The O4:K12 serotype exhibited the highest prevalence among tested clinical *V. parahaemolyticus* isolates from the U.S. Pacific Coast between 1979 and 1995 (Depaola et al., 2003).

Between May and June 1998, 416 individuals in 13 states reported experiencing gastroenteritis after having eaten raw oysters harvested from Galveston Bay, Tex. All of the 28 available stool samples yielded *V. parahaemolyticus* O3:K6 isolates, which closely resembled the pandemic Asian O3:K6 isolates by PFGE (Daniels et al., 2000a). During July to September 1998, a smaller outbreak of O3:K6 *V. parahaemolyticus* associated with the consumption of oysters and clams harvested from Long Island Sound occurred among residents of Connecticut, New Jersey, and New York (CDC, 1999a,b). Interestingly, prior to 1998, outbreaks of the O3:K6 *V. parahaemolyticus* serotype had never been reported in the U.S. (Daniels et al., 2000a). *V. parahaemolyticus* has also been detected as far north as Alaska (Vasconcelos et al., 1975). Active surveillance identified a total of 62 patients with gastroenteritis; the outbreak was associated with the O6:K18 serotype, which was responsible for a 30% infection rate among passengers consuming one to six oysters on three cruises over a two-week period in July 2004 (McLaughlin et al., 2005). The O6:K18 isolates from the Alaskan outbreak were indistinguishable by PFGE from those isolated in the sporadic cases from Pacific Coast states over the previous decade. From July to October of 2004, 96 environmental samples (e.g., oysters, water, and sediment) were collected from 17 Alaskan oyster farms, and 31 samples (32 percent) tested positive for *V. parahaemolyticus*. The most frequently occurring serotypes were O1:K9, O4:K63, and O6:K18 (Newton et al., 2012) (Figure 1 and Table 1).

Clinical isolates in the U.S., especially from the Pacific Northwest also encode *trh* in addition to *tdh* (Paranjpye et al., 2012). An increasing proportion of clinical isolates possessed neither *tdh* nor *trh* genes, and these were associated with the most severe cases that required hospitalization (FAO/WHO, 2011).

Due to elevated prevalence of *Vibrio* infection, the following two national surveillance systems have been created to monitor the cases of infection: the national Cholera and Other *Vibrio* Illness Surveillance (COVIS) system and the Foodborne Diseases Active Surveillance Network (Food-Net). COVIS was established in 1988 by the CDC; the Gulf Coast states of Alabama, Florida, Louisiana, and Texas (states with high incidences of vibriosis); and the US Food and Drug Administration (FDA) to conduct surveillance of illnesses caused by *Vibrio* species (CDC, 2013a). FoodNet is a collaborative project that includes the CDC, 10 participating state health departments, the Food Safety and Inspection Service of the US Department of Agriculture, and the FDA. FoodNet conducts active, population-based surveillance for all laboratory-confirmed Vibrio infections, as well as other enteric infections that are commonly transmitted through food (CDC, 2013b).

### Brazil

In relation to Brazil, there are few references to *V. parahaemolyticus* in cases of intestinal infections of human isolates (Hofer, 1983; Magalhaes et al., 1991a,b, 1992) in the colonization of cutaneous wounds (Rodrigues et al., 2001). In 1991 in Recife, a tropical city situated in Northeast Brazil, *V. parahaemolyticus* cultures were isolated from 21 cases of gastroenteritis associated with the consumption of different types of seafood. The serotypes detected belonged to diverse serovars, including O1:K56, O3:K5, O3:K58, O3:KUT, O4:K4,O4:K10, O4:K12, O4:K53, O4:KUT, O5:KUT, and O10:KUT (Magalhaes et al., 1991a). In 1992, *V. parahaemolyticus* strains were identified in 18 human isolates and found to carry either the *tdh*, *trh*, or both genes (Magalhaes et al., 1992). Until 2001–2002, the presence of strains with the characteristics of the pandemic clone (O3:K6) was detected in two outbreaks of gastroenteritis, suggesting that this strain is disseminated in certain regions (Ceara and Alagoas) of northeast Brazil. Nonetheless, the O3:KUT serotype of *V. parahaemolyticus* was detected in this region (Pernambuco and Alagoas) at the same time (Magalhaes et al., 1992) (Figure 1 and Table 1).

In 2010, studies on oysters harvested in the Southern coastal areas of Sao Paulo, Brazil found that the 99.2–100% contained *V. parahaemolyticus* (Sobrinho Pde et al., 2010, 2011). The results of these studies indicated that the levels of *V. parahaemolyticus* in retail oysters are frequently above 10,000 cfu/g, but the pathogenic strains are infrequent (none of the isolates contained the *tdh* and/or *trh* genes) (Sobrinho Pde et al., 2010, 2011). This corroborates the limited number of reported outbreaks, as only three *V. parahaemolyticus* outbreaks were reported between 1999 and 2009, two in 2003 and one in 2006, affecting 39 patients (Sobrinho Pde et al., 2010, 2011). However, because of the virulence potential of the pandemic clone detected at northeast Brazil in 2001–2002, increased seawater temperature and the consumption of seafood (oysters and mussels) represent factors that are extremely favorable for the dissemination of this microorganism.

### Mexico

In 1993, the prevalence of *V. parahaemolyticus* in fresh seafood sold in a region of Mexico was 45.6%, of which 71.4% was fish, 44% was oysters and 27.6% was shrimp. Of the total seafood sold, a larger number of positive samples was observed during the warmer months (Torres-Vitela and Fernandez-Escartin, 1993). Between September and October of 2004, more than 1230 cases of gastroenteritis were reported in the relatively small geographical area of the southern part of Sinaloa, a state located in Northwest Mexico.

These cases were associated with the consumption of raw or undercooked shrimp that were collected in a lagoon system. In Mexico, this was the first outbreak of gastroenteritis that was caused by the pandemic strain of O3:K6 *V. parahaemolyticus* (Cabanillas-Beltran et al., 2006; Velazquez-Roman et al., 2012). Prior to 2004, there had been no reports of outbreaks caused by *V. parahaemolyticus* in Mexico (Cabanillas-Beltran et al., 2006; Velazquez-Roman et al., 2012) and few reports of environmental *tdh*-positive strains had been isolated from water, fish, sediment and shrimp (Cabrera-Garcia et al., 2004; Velazquez-Roman et al., 2012). Since 2004, recurrent sporadic cases have been reported in this state. During recent years, new cases have arisen in different areas of Sinaloa (including the southern and northern areas). Data analyzed over 6 years (from September 2004 to December 2010) in clinical cases and environmental samples along the Pacific coast of Sinaloa detected the presence of pandemic and pathogenic strains belonging to different serotypes of *Vibrio parahaemolyticus* (Figure 1 and Table 1). The pandemic O3:K6 clone was the most prevalent serotype isolated from clinical samples during every year of the study. The pandemic strain was also detected in environmental samples (seawater, sediment, and shrimp), suggesting that it is endemic to this setting (Velazquez-Roman et al., 2012).

To date, a shift in the epidemiology of outbreaks caused by pandemic *V. parahaemolyticus* O3:K6 has not been established in Mexico, where 80.5% of clinical cases were caused by this pathogen. However, the data from 2010/2011 in Chile showed that the pandemic O3:K6 strain has practically disappeared and that this condition is associated with a change in serotype of many pandemic isolates to O3:K59, suggesting the emergence of new clinical strains (Harth et al., 2009; Garcia et al., 2013). Thus, on the American continent, the last report of the O3:K6 pandemic clone associated with clinical cases was observed in Mexico.

## *vibrio parahaemolyticus* in countries without the pandemic american continent clone

### Canada

In 1997–2006, 212 cases of gastroenteritis were reported in British Columbia and were based predominantly on laboratory confirmation (*Vibrio parahaemolyticus*), with a minority of cases based on an association of clinical symptoms and food history (Figure 1 and Table 1). From 2001 to 2006, *V. parahaemolyticus* was the most commonly reported Vibrio species, and the majority of *V. parahaemolyticus* infections are believed to have resulted from the consumption of raw shellfish (in particular, raw oysters) (CDC, 2008). The majority of *V. parahaemolyticus* infections occurred during the summer months when ocean temperatures are warmer (PHAC, 2013). Regardless of the underlying reason, these findings suggest the need to increase awareness of the risk of *Vibrio* infection from shellfish (PHAC, 2013). Considering the increased rate of *V. parahaemolyticus* infection in 2006, the BC Center for Disease Control, BC health authorities, Health Canada, the CFIA (2007), the BC Shellfish Growers Association and the Canadian Council of Grocery Distributors have developed an education and communication plan to increase awareness for the risks associated with consuming raw shellfish (Khaira and Galanis, 2007).

### Costa rica

From November 1985 to October 1987, *Vibrio parahaemolyticus* and other *Vibrios* were detected in 36 sediment samples and 41 bivalve samples obtained from 3 collection sites in the Gulf of Nicoya, Costa Rica (Garcia Cortes and Antillon, 1990). However, the first reported case of diarrhea attributed to *V. parahaemolyticus* was isolated in 1998 in a 30-year-old man in Costa Rica (Díaz, 2002), after consuming “pianguas” (*Anadara tuberculosa*), he presented severe and watery diarrhea, vomiting and abdominal cramps. No additional data are available for this pathogen in Costa Rica (Figure 1 and Table 1).

### Puerto rico

In 1988, water and shellfish samples collected from estuaries, mangroves, and beaches along the coast of Puerto Rico were examined for *V. vulnificus* and *V. parahaemolvticus* (Rivera Susan Lugo and Hazen, 1989) (Table 1). That study demonstrated a significant positive correlation between fecal coliform levels and the density of *V. parahaemolyticus* in the water column, although no other reports are available for this bacterium.

### Panama

In 1974, the presence of *V. parahaemolyticus* was detected in seawater off the Pacific coast of Panama (Kourany et al., 1974), and in 1975 (Kourany and Vasquez, 1975) the first confirmed case of gastroenteritis attributed to *Vibrio parahaemolyticus* infection in Panama was reported. Beside, *Vibrio parahaemolyticus* was recovered from fresh shrimp used in preparing seafood dishes (Kourany and Vasquez, 1975) (Figure 1 and Table 1). However, no other data regarding the isolates of *V. parahaemolyticus* to date have been reported.

### Venezuela

In Venezuela, *V. parahaemolyticus* has been isolated from bivalves (*Arca zebra* and *Perna perna*) in an area near the Cariaco Basin (Grau et al., 2004; Muñoz et al., 2008). In 2011, *Vibrio parahaemolyticus* was detected in the prawns and corals of this system (Garcia-Amado et al., 2011) (Figure 1 and Table 1). However, additional data are unavailable for this pathogen.

### Argentina

The only accessible data regarding the detection of *V. parahemolyticus* in this country is a report in 1977, when *V. parahemolyticus* was isolated from mussel varieties (Casellas et al., 1977) (Figure 1 and Table 1). However, no additional data are available for this region.

## The dissemination of *vibrio parahaemolyticus* O3:K6 on the american continent

How and when the pandemic strain arrived in these countries and regions of the American continent and why it caused the outbreaks during those years remains a matter for speculation. The routes and mechanisms underlying the dissemination of the pandemic O3:K6 clone have been controversial from their emergence, although biological invasion by *Vibrio* populations can be mediated by human activities, such as ballast water discharges, or induced by natural events, such as the movement of oceanic waters.

Until the emergence of *Vibrio* epidemics in South America, the infections had spread predominantly westward, consistent with the prevailing westward movement of water associated with the Indonesian throughflow (a system of currents flowing from the Pacific Ocean to the Indian Ocean through the Indonesian Sea) (Gordon et al., 2003). The infections first surfaced in the north of the country and then spread southwards along more than 1500 km within a 4-month period until they reached the Chilean city of Antofagasta (Gonzalez-Escalona et al., 2005). The origins and routes of dissemination of pandemic *V. parahaemolyticus* from its arrival in South America remain unknown. A recent revision of the oceanographic conditions during this period revealed that the emergence and dissemination of the pandemic clone in Peru correlated with the dynamics of the progression and recession of the 1997 El Niño waters (Martinez-Urtaza et al., 2008). According to this study, the 1997 El Niño episode might have provided an extraordinary corridor for the displacement of Asian *Vibrio* populations to America, causing a general disruption of the environmental conditions of coastal areas and the southward displacement of native species (Ansede-Bermejo et al., 2010). In addition to promoting natural range expansions, warming temperatures could facilitate the establishment and spread of deliberately or accidentally introduced species (Carlton, 2000; Stachowicz et al., 2002). The chance of success of a biological invasion positively correlates with the level of ecological disturbance of the invaded environment (Lockwood et al., 2005).

One of the most recurrent explanations has been based on the discharge of ballast waters from ships traveling from areas of *V. parahaemolyticus* endemicity (Ansede-Bermejo et al., 2010). Ballast water discharges have been considered one of the major vehicles for the worldwide dissemination of marine species and biological invasions (Niimi, 2004), and they have been identified as a reliable mechanism for the propagation of pathogenic *Vibrios* (Depaola et al., 1992; McCarthy and Khambaty, 1994; Ruiz et al., 2000). Discharge of ballast waters should be related to a low propagule pressure or introductory effort as follows: a unique introduction event of reduced genetic variation with low chances of success of establishment within the invaded area (Lockwood et al., 2005). Ballast water-mediated invasions have been proposed for the introduction of the O3:K6 strains into Texas in 1998 (Daniels et al., 2000b). The infections caused a unique epidemic outbreak in areas close to ports, although after the outbreaks, the strains belonging to the O3:K6 clone were never detected in the clinical cases or the environment (Depaola et al., 2000). Bacterial dispersal through ballast water, however, fails to consistently and comprehensively explain the emergence of some epidemic episodes of *V. parahaemolyticus* (Ansede-Bermejo et al., 2010). This is particularly true where infections have emerged and spread rapidly over hundreds of kilometers of coastline, as in the case of the arrival, dissemination and establishment of the O3:K6 clone populations along the Pacific Coast of South America (Peru) since 1996 (Leal et al., 2008; Martinez-Urtaza et al., 2008).

The importance of water temperature in the epidemiology of infections is reflected by the fact that most outbreaks have occurred during the warmer months of the year. Thus, the spread or arrival of O3:K6 to Brazil in 2001 may be attributed to the increased seawater temperature, a factor that is extremely favorable for the dissemination of this microorganism. However, how this bacterium spread to Brazil remains to be determined. Warmer temperatures also appear to extend the geographical range of *V. parahaemolyticus* into areas such as Alaska (McLaughlin et al., 2005) and Chile (Gonzalez-Escalona et al., 2005), whereas the restoration of cold waters into these areas may have begun to reduce the O3:K6 populations toward the tropical areas located in northern Peru, where they remain endemic to date (Martinez-Urtaza et al., 2008). Alaska is located 1000 km further north than any previously reported *V. parahaemolyticus* illnesses. The unprecedentedly high prevalence of pathogenic strains of *V. parahaemolyticus* among the population of Alaskan oysters, and perhaps their increased virulence compared to other pathogenic strains, was particularly unexpected and remains unexplained (McLaughlin et al., 2005). Although oysters are the most common food associated with *Vibrio* infection in certain countries (Hlady and Klontz, 1996), there have been reports of *V. parahaemolyticus* infections associated with other types of seafood (Velazquez-Roman et al., 2012). Climate warming can increase pathogen development and survival rates, disease transmission and host vulnerability, although a subset of pathogens might decrease with warming, releasing hosts from disease (Harvell et al., 2002).

Climate change might also influence the selection of different modes of transmission and virulence (Marcogliese, 2001). Recently, analysis of the arrival of the O3:K6 clone at the Pacific coasts of South America has provided novel insights linking its origin to an invasion in 1996 from the Asian (Indian) populations and describing the successful establishment of O3:K6 populations, first in Peru and subsequently in the south of Chile (1998) (Ansede-Bermejo et al., 2010). The Peruvian foothold of the O3:K6 clone may be the origin of the arrival of this strain to the coasts of Northwestern Mexico (Sinaloa) in 2003 to 2004, traveling more than 8000 Km due to either ballast water discharge or climate change (Velazquez-Roman et al., 2012). However, the actual dissemination dynamics of the environmental human pathogen *Vibrio parahaemolyticus* remain uncertain. El Niño provides a partial explanation for the spectrum of marine biotoxins (red tides) on the coast of Mexico (Ochoa, 2003).Notwithstanding, the absence of environmental information about the impact of the arrival of El Niño waters on the native *Vibrio* community severely limits a comprehensive overview of the role of El Niño episodes in the introduction of new *Vibrio* pathogens into these remote regions. All of these factors, including climate change and human activities, may have influenced and favored the dissemination of the O3:K6 pandemic clone and its serovariants to American countries.

Nevertheless, pathogenic subpopulations of Vibrios are potential agents of disease outbreaks and pandemics (Siddique et al., 1994; CDC, 1999b; Depaola et al., 2000; Lee et al., 2001; Gonzalez-Escalona et al., 2005; McLaughlin et al., 2005; Noriea et al., 2010). The origin and subsequent spread of the O3:K6 isolates of *V. parahaemolyticus* must be the consequence of coincidental events that varied in magnitude in developing countries, particularly on the American continent, where the consumption of raw or undercooked shellfish is common (Wong et al., 1999; Chen et al., 2004; Velazquez-Roman et al., 2012) but varies according to prevailing levels of health education, sanitation, risk factors, response from the community at large and climate change (Ansede-Bermejo et al., 2010).

## Conclusions

On the American continent, only five countries have reported the presence of the pandemic *Vibrio parahaemolyticus* clone O3:K6 and its serovariants in clinical cases and/or the environment. The pandemic strain was first observed in Peru (1996), subsequently spread to Chile in 1998, to the U.S. in 1998, to Brazil in 2001 and to Mexico in 2004. However, the presence of pandemic clones and the number of reported outbreaks in the countries of the American continent may be underestimated due to non-reporting. The American continent has a diversified climate. The scientific communities are coming to the conclusion that ballast discharge, global trade and climate change represent the major underlying mechanisms for the global spread of pandemic *V. parahaemolyticus*, particularly clone O3:K6. Moreover, many American countries require active reporting, diagnosis, and surveillance of cases and contacts, food handlers, and sanitation, as well as health education programs for the prevention of gastroenteritis due to *V. parahaemolyticus*.

### Conflict of interest statement

The authors declare that the research was conducted in the absence of any commercial or financial relationships that could be construed as a potential conflict of interest.
